# Poly[tri-μ-aqua-di­aqua-μ-phosphono­formato-cobalt(II)sodium]

**DOI:** 10.1107/S1600536813012853

**Published:** 2013-05-18

**Authors:** Xu-Jian Luo, Xiu-Qing Zhang

**Affiliations:** a College of Chemistry and Chemical Engineering, Central South University, Changsha 410083, People’s Republic of China; b College of Chemistry and Bioengineering, Guilin University of Technology, Guilin 541004, People’s Republic of China

## Abstract

The title complex, [CoNa(CO_5_P)(H_2_O)_5_]_n_, was obtained by reacting sodium phosphono­formate with cobalt nitrate. The complex contains cobalt(II) and sodium ions, which are bridged by the O atoms of two aqua ligands. The Co^II^ ion is octahedrally coordinated by three phosphonoformato ligands (one bi- and the other monodentate) and by two O atoms from the bridging aqua ligands. The sodium cation is hexa­coordinated by six O atoms from four bridging and two terminal aqua ligands. The complex mol­ecules are linked to give a three-dimensional structure by phosphono­formate ligands bridging Co^II^ atoms and water mol­ecules establishing cobalt–sodium bridges. O—H⋯O hydrogen bonding between the aqua ligands and all O atoms of the phosphono­formato ligand and neighbouring aqua ligands help to consolidate the packing.

## Related literature

For biological applications of organo­phospho­rus complexes, see: Xue *et al.* (2010 ▶); Torres Martin de Rosales *et al.* (2009 ▶); Galanski *et al.* (2003 ▶); Margiotta *et al.* (2007 ▶); Mesri *et al.* (1996 ▶). 

## Experimental

### 

#### Crystal data

[CoNa(CO_5_P)(H_2_O)_5_]
*M* *_r_* = 294.98
Monoclinic, 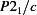

*a* = 8.299 (2) Å
*b* = 11.785 (3) Å
*c* = 9.769 (3) Å
β = 106.204 (4)°
*V* = 917.5 (4) Å^3^
*Z* = 4
Mo *K*α radiation
μ = 2.13 mm^−1^
*T* = 223 K
0.30 × 0.14 × 0.05 mm

#### Data collection

Rigaku Saturn diffractometer
Absorption correction: multi-scan (*REQAB*; Jacobson, 1998 ▶) *T*_min_ = 0.636, *T*_max_ = 0.899
3715 measured reflections
1691 independent reflections
1539 reflections with *I* > 2σ(*I*)
*R*_int_ = 0.028

#### Refinement

*R*[*F* ^2^ > 2σ(*F* ^2^)] = 0.035
*wR*(*F* ^2^) = 0.066
*S* = 1.09
1691 reflections
168 parameters
10 restraints
All H-atom parameters refined
Δρ_max_ = 0.37 e Å^−3^
Δρ_min_ = −0.45 e Å^−3^

### 

Data collection: *CrystalClear* (Rigaku, 1999 ▶); cell refinement: *CrystalClear*; data reduction: *CrystalStructure* (Rigaku/MSC & Rigaku, 2000 ▶); program(s) used to solve structure: *SHELXS97* (Sheldrick, 2008 ▶); program(s) used to refine structure: *SHELXL97* (Sheldrick, 2008 ▶); molecular graphics: *SHELXTL* (Sheldrick, 2008 ▶); software used to prepare material for publication: *publCIF* (Westrip, 2010 ▶).

## Supplementary Material

Crystal structure: contains datablock(s) I, global. DOI: 10.1107/S1600536813012853/im2427sup1.cif

Structure factors: contains datablock(s) I. DOI: 10.1107/S1600536813012853/im2427Isup2.hkl

Additional supplementary materials:  crystallographic information; 3D view; checkCIF report

## Figures and Tables

**Table 1:** Hydrogen-bond geometry (Å, °)

*D*—H⋯*A*	*D*—H	H⋯*A*	*D*⋯*A*	*D*—H⋯*A*
O10—H10*B*⋯O4^i^	0.82 (1)	1.99 (1)	2.805 (3)	173 (4)
O10—H10*A*⋯O5^ii^	0.82 (1)	1.99 (3)	2.736 (3)	151 (5)
O9—H9*B*⋯O1^iii^	0.82 (1)	1.87 (1)	2.683 (3)	178 (4)
O9—H9*A*⋯O5^iv^	0.82 (1)	1.98 (1)	2.790 (4)	171 (4)
O8—H8*B*⋯O4^v^	0.82 (1)	2.24 (2)	2.988 (4)	152 (3)
O8—H8*A*⋯O2^iii^	0.82 (1)	1.94 (1)	2.751 (3)	168 (4)
O7—H7*B*⋯O10^vi^	0.82 (1)	1.88 (1)	2.703 (4)	177 (4)
O7—H7*A*⋯O5^ii^	0.82 (1)	1.96 (2)	2.757 (3)	166 (5)
O6—H6*B*⋯O8^iii^	0.82 (1)	2.02 (2)	2.806 (3)	161 (4)
O6—H6*A*⋯O3^ii^	0.82 (1)	1.97 (2)	2.698 (3)	148 (4)

Symmetry codes: (i) 
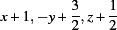
; (ii) 
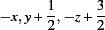
; (iii) 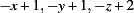; (iv) 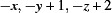; (v) 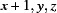; (vi) 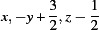.

**Fig. 1.**
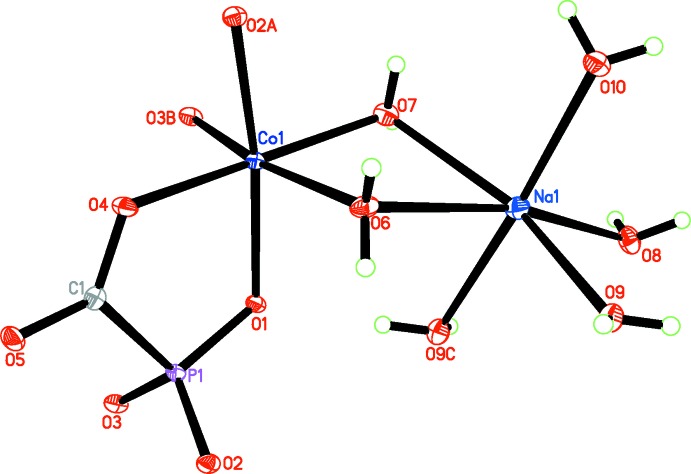
Molecular structure of one repeating unit of the coordination polymer with the atom-numbering scheme displacement ellipsoids drawn at the 30% probability level.

**Fig. 2.**
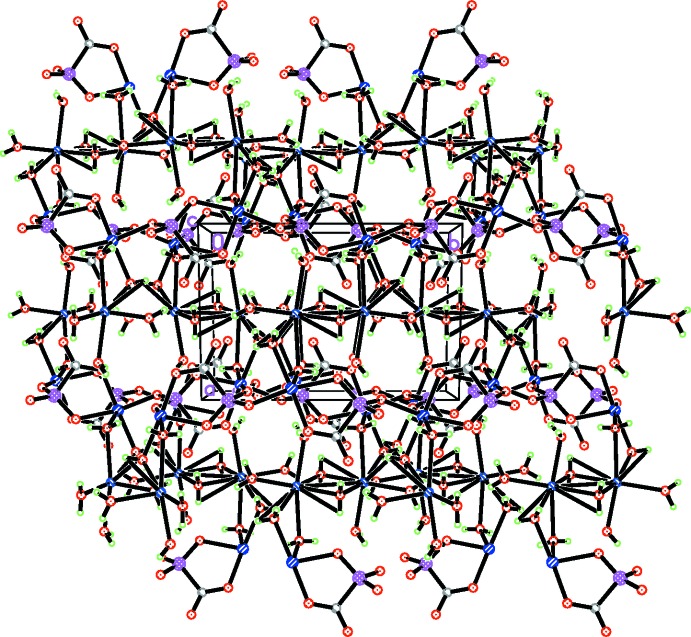
Packing diagram of the title compound viewed along c axis.

## supplementary crystallographic information

### Comment

Organophosphates have been widely used in medicinal chemistry and life science. They play an important role in life processes of substance transportation and energy transformation, and are also important for biological substances, such as ATP, DNA, RNA, *etc*. Bisphosphonates (BPs) are metabolically stable analogues of pyrophosphates. They have a very high affinity to calcium ions and therefore show a very strong inhibitory effect on osteoclastic resorption. They are used as therapeutic agents for several bone-related diseases. Foscarnet and phosphonoacetic acid are known to inhibit viral DNA polymerase, inhibit the replication of herpes viruses, and also inhibit retroviruses (Mesri *et al.*, 1996). Recently, several bifunctional metal-phosphonate complexes have been explored (Galanski *et al.*, 2003; Margiotta *et al.*, 2007; Xue *et al.*, 2010; Torres Martin de Rosales *et al.*, 2009).

The molecular structure of the title compound is shown in Fig. 1. Each Co(II) ion is in an octahedral environment coordinated by two O atoms (O1, O4) from a chelating phosphonoformate ligand, two O atoms (O6, O7) from two bridging water molecules and two O atoms (O2A, O3B) from two other phosphonoformates. Similarly, the Na(I) ion is coordinated by four O atoms (O6, O7, O8, O9) from four bridging water molecules and two O atoms (O9C, O10) from two terminal water ligands. The complex is linked to 3-D structure by phosphonoformate ligands bridging cobalt atoms and water molecules establishing cobalt sodium bridges (Fig. 2).

### Experimental

An aqueous solution (15 ml) of Co(NO_3_)_2_ × 6 H_2_O (0.145 g, 0.5 mmol) was added dropwisely to an aqueous solution (15 ml) of sodium phosphonoformate (0.180 g, 0.6 mmol) at 323 K. The resulting mixture was refluxed for 3 h, and then the aqueous solution was allowed to cool down to room temperature. Pink block shaped crystals suitable for X-ray single diffraction analysis were harvested by slow evaporation (yield, 65%).

### Refinement

H atoms of the water molecules were located in a difference Fourier map and refined, with O—H distances restrained to 0.82 Å.

### Crystal data

**Table:**

[CoNa(CO_5_P)(H_2_O)_5_]	*F*(000) = 596
*M**_r_* = 294.98	*D*_x_ = 2.135 Mg m^−^^3^
Monoclinic, *P*2_1_/*c*	Mo *K*α radiation, λ = 0.71075 Å
Hall symbol: -P 2ybc	Cell parameters from 3028 reflections
*a* = 8.299 (2) Å	θ = 3.1–27.5°
*b* = 11.785 (3) Å	µ = 2.13 mm^−^^1^
*c* = 9.769 (3) Å	*T* = 223 K
β = 106.204 (4)°	Block, pink
*V* = 917.5 (4) Å^3^	0.30 × 0.14 × 0.05 mm
*Z* = 4	

### Data collection

**Table:**

Rigaku Saturn diffractometer	1691 independent reflections
Radiation source: fine-focus sealed tube	1539 reflections with *I* > 2σ(*I*)
Graphite monochromator	*R*_int_ = 0.028
Detector resolution: 14.63 pixels mm^-1^	θ_max_ = 25.5°, θ_min_ = 3.1°
ω scans	*h* = −8→10
Absorption correction: multi-scan (*REQAB*; Jacobson, 1998)	*k* = −12→14
*T*_min_ = 0.636, *T*_max_ = 0.899	*l* = −11→7
3715 measured reflections	

### Refinement

**Table:**

Refinement on *F*^2^	Primary atom site location: structure-invariant direct methods
Least-squares matrix: full	Secondary atom site location: difference Fourier map
*R*[*F*^2^ > 2σ(*F*^2^)] = 0.035	Hydrogen site location: inferred from neighbouring sites
*wR*(*F*^2^) = 0.066	All H-atom parameters refined
*S* = 1.09	*w* = 1/[σ^2^(*F*_o_^2^) + (0.0277*P*)^2^] where *P* = (*F*_o_^2^ + 2*F*_c_^2^)/3
1691 reflections	(Δ/σ)_max_ = 0.001
168 parameters	Δρ_max_ = 0.37 e Å^−^^3^
10 restraints	Δρ_min_ = −0.45 e Å^−^^3^

### Special details

**Table:**

Geometry. All e.s.d.'s (except the e.s.d. in the dihedral angle between two l.s. planes) are estimated using the full covariance matrix. The cell e.s.d.'s are taken into account individually in the estimation of e.s.d.'s in distances, angles and torsion angles; correlations between e.s.d.'s in cell parameters are only used when they are defined by crystal symmetry. An approximate (isotropic) treatment of cell e.s.d.'s is used for estimating e.s.d.'s involving l.s. planes.
Refinement. Refinement of *F*^2^ against ALL reflections. The weighted *R*-factor *wR* and goodness of fit *S* are based on *F*^2^, conventional *R*-factors *R* are based on *F*, with *F* set to zero for negative *F*^2^. The threshold expression of *F*^2^ > σ(*F*^2^) is used only for calculating *R*-factors(gt) *etc*. and is not relevant to the choice of reflections for refinement. *R*-factors based on *F*^2^ are statistically about twice as large as those based on *F*, and *R*- factors based on ALL data will be even larger.

### Fractional atomic coordinates and isotropic or equivalent isotropic displacement parameters (Å^2^)

**Table:**

	*x*	*y*	*z*	*U*_iso_*/*U*_eq_	
Co1	0.08112 (5)	0.64526 (3)	0.69444 (4)	0.01133 (13)	
P1	−0.00606 (10)	0.38705 (6)	0.65570 (8)	0.01079 (19)	
Na1	0.49306 (16)	0.63218 (10)	0.95685 (13)	0.0201 (3)	
O1	0.1359 (3)	0.47211 (16)	0.6737 (2)	0.0137 (5)	
O2	0.0139 (3)	0.30646 (17)	0.7807 (2)	0.0146 (5)	
O3	−0.0498 (3)	0.32183 (16)	0.5160 (2)	0.0147 (5)	
O4	−0.1606 (3)	0.58356 (16)	0.6816 (2)	0.0158 (5)	
O5	−0.3256 (3)	0.43235 (17)	0.6558 (2)	0.0189 (5)	
O6	0.1718 (3)	0.62170 (19)	0.9232 (2)	0.0179 (5)	
H6A	0.139 (5)	0.668 (3)	0.971 (4)	0.047 (14)*	
H6B	0.164 (4)	0.5600 (16)	0.960 (3)	0.024 (10)*	
O7	0.3298 (3)	0.71602 (19)	0.7377 (3)	0.0171 (5)	
H7A	0.313 (6)	0.777 (2)	0.770 (5)	0.088 (19)*	
H7B	0.388 (4)	0.706 (3)	0.683 (3)	0.029 (11)*	
O8	0.7866 (3)	0.6010 (2)	0.9711 (3)	0.0226 (6)	
H8A	0.850 (4)	0.636 (3)	1.038 (3)	0.048 (14)*	
H8B	0.816 (4)	0.619 (3)	0.901 (2)	0.027 (11)*	
O9	0.5412 (3)	0.5383 (2)	1.1731 (3)	0.0189 (5)	
H9A	0.473 (3)	0.540 (3)	1.220 (3)	0.031 (12)*	
H9B	0.639 (2)	0.537 (3)	1.221 (4)	0.040 (13)*	
O10	0.5291 (3)	0.8103 (2)	1.0633 (3)	0.0219 (6)	
H10A	0.484 (7)	0.866 (3)	1.017 (5)	0.12 (2)*	
H10B	0.620 (3)	0.839 (3)	1.104 (4)	0.043 (13)*	
C1	−0.1872 (4)	0.4775 (2)	0.6624 (3)	0.0133 (7)	

### Atomic displacement parameters (Å^2^)

**Table:**

	*U*^11^	*U*^22^	*U*^33^	*U*^12^	*U*^13^	*U*^23^
Co1	0.0135 (2)	0.0099 (2)	0.0113 (2)	−0.00024 (18)	0.00465 (17)	0.00001 (17)
P1	0.0130 (4)	0.0095 (4)	0.0109 (4)	0.0001 (3)	0.0048 (3)	0.0003 (3)
Na1	0.0223 (7)	0.0168 (6)	0.0199 (7)	0.0021 (6)	0.0037 (6)	−0.0001 (5)
O1	0.0123 (11)	0.0095 (10)	0.0190 (12)	−0.0013 (9)	0.0040 (9)	0.0003 (9)
O2	0.0205 (12)	0.0125 (10)	0.0101 (11)	−0.0016 (9)	0.0030 (9)	0.0013 (9)
O3	0.0230 (13)	0.0117 (10)	0.0101 (11)	−0.0018 (9)	0.0055 (10)	−0.0005 (9)
O4	0.0170 (12)	0.0103 (10)	0.0222 (12)	−0.0006 (9)	0.0090 (10)	−0.0009 (9)
O5	0.0135 (12)	0.0158 (11)	0.0286 (14)	−0.0032 (10)	0.0078 (10)	0.0000 (10)
O6	0.0261 (14)	0.0141 (12)	0.0152 (12)	0.0033 (11)	0.0085 (11)	0.0011 (11)
O7	0.0197 (14)	0.0171 (12)	0.0173 (13)	0.0003 (11)	0.0096 (11)	−0.0026 (10)
O8	0.0244 (14)	0.0275 (13)	0.0157 (14)	−0.0062 (11)	0.0054 (12)	−0.0047 (12)
O9	0.0153 (14)	0.0238 (13)	0.0187 (14)	0.0017 (11)	0.0065 (12)	0.0003 (10)
O10	0.0221 (15)	0.0181 (12)	0.0250 (15)	−0.0031 (11)	0.0059 (12)	−0.0010 (11)
C1	0.0145 (17)	0.0158 (16)	0.0098 (17)	0.0013 (14)	0.0038 (14)	−0.0018 (13)

### Geometric parameters (Å, º)

**Table:**

Co1—O3^i^	2.036 (2)	Na1—H7A	2.64 (4)
Co1—O2^ii^	2.097 (2)	O2—Co1^iv^	2.097 (2)
Co1—O4	2.104 (2)	O3—Co1^i^	2.036 (2)
Co1—O1	2.113 (2)	O4—C1	1.274 (3)
Co1—O7	2.156 (3)	O5—C1	1.251 (4)
Co1—O6	2.168 (2)	O6—H6A	0.819 (10)
P1—O1	1.519 (2)	O6—H6B	0.820 (10)
P1—O2	1.519 (2)	O7—H7A	0.817 (10)
P1—O3	1.520 (2)	O7—H7B	0.820 (10)
P1—C1	1.859 (3)	O8—H8A	0.821 (10)
Na1—O9	2.319 (3)	O8—H8B	0.817 (10)
Na1—O10	2.325 (3)	O9—Na1^iii^	2.351 (3)
Na1—O9^iii^	2.351 (3)	O9—H9A	0.822 (10)
Na1—O7	2.404 (3)	O9—H9B	0.818 (10)
Na1—O8	2.429 (3)	O10—H10A	0.822 (10)
Na1—O6	2.596 (3)	O10—H10B	0.818 (10)
Na1—Na1^iii^	3.221 (2)		
			
O3^i^—Co1—O2^ii^	89.89 (8)	O8—Na1—Na1^iii^	82.90 (8)
O3^i^—Co1—O4	98.89 (9)	O6—Na1—Na1^iii^	87.00 (7)
O2^ii^—Co1—O4	86.39 (8)	O9—Na1—H7A	149.2 (10)
O3^i^—Co1—O1	93.29 (8)	O10—Na1—H7A	72.6 (5)
O2^ii^—Co1—O1	169.80 (9)	O9^iii^—Na1—H7A	102.7 (5)
O4—Co1—O1	83.54 (8)	O7—Na1—H7A	17.9 (5)
O3^i^—Co1—O7	88.26 (9)	O8—Na1—H7A	120.2 (11)
O2^ii^—Co1—O7	89.84 (9)	O6—Na1—H7A	65.1 (11)
O4—Co1—O7	171.90 (9)	Na1^iii^—Na1—H7A	141.2 (8)
O1—Co1—O7	99.94 (9)	P1—O1—Co1	117.89 (13)
O3^i^—Co1—O6	167.17 (9)	P1—O2—Co1^iv^	134.36 (12)
O2^ii^—Co1—O6	91.74 (8)	P1—O3—Co1^i^	137.88 (13)
O4—Co1—O6	93.92 (9)	C1—O4—Co1	118.1 (2)
O1—Co1—O6	87.32 (8)	Co1—O6—Na1	99.98 (10)
O7—Co1—O6	79.02 (9)	Co1—O6—H6A	116 (3)
O1—P1—O2	114.36 (12)	Na1—O6—H6A	113 (3)
O1—P1—O3	114.88 (13)	Co1—O6—H6B	121 (2)
O2—P1—O3	110.56 (12)	Na1—O6—H6B	101 (2)
O1—P1—C1	103.06 (13)	H6A—O6—H6B	105 (4)
O2—P1—C1	103.76 (14)	Co1—O7—Na1	106.63 (11)
O3—P1—C1	109.21 (12)	Co1—O7—H7A	99 (4)
O9—Na1—O10	93.19 (10)	Na1—O7—H7A	98 (3)
O9—Na1—O9^iii^	92.76 (10)	Co1—O7—H7B	121 (3)
O10—Na1—O9^iii^	173.87 (11)	Na1—O7—H7B	103 (2)
O9—Na1—O7	156.76 (11)	H7A—O7—H7B	125 (4)
O10—Na1—O7	89.86 (9)	Na1—O8—H8A	112 (3)
O9^iii^—Na1—O7	85.24 (9)	Na1—O8—H8B	116 (3)
O9—Na1—O8	87.73 (10)	H8A—O8—H8B	105 (4)
O10—Na1—O8	96.27 (10)	Na1—O9—Na1^iii^	87.24 (10)
O9^iii^—Na1—O8	82.52 (10)	Na1—O9—H9A	122 (3)
O7—Na1—O8	114.84 (10)	Na1^iii^—O9—H9A	109 (3)
O9—Na1—O6	90.14 (9)	Na1—O9—H9B	115 (3)
O10—Na1—O6	95.69 (9)	Na1^iii^—O9—H9B	104 (3)
O9^iii^—Na1—O6	85.74 (9)	H9A—O9—H9B	114 (4)
O7—Na1—O6	66.63 (9)	Na1—O10—H10A	119 (4)
O8—Na1—O6	167.95 (9)	Na1—O10—H10B	125 (3)
O9—Na1—Na1^iii^	46.79 (7)	H10A—O10—H10B	99 (4)
O10—Na1—Na1^iii^	139.96 (10)	O5—C1—O4	123.0 (3)
O9^iii^—Na1—Na1^iii^	45.97 (7)	O5—C1—P1	119.6 (2)
O7—Na1—Na1^iii^	126.93 (8)	O4—C1—P1	117.3 (2)
			
O2—P1—O1—Co1	−112.78 (14)	O7—Na1—O6—Co1	20.00 (8)
O3—P1—O1—Co1	117.81 (14)	O8—Na1—O6—Co1	−79.7 (5)
C1—P1—O1—Co1	−0.87 (16)	Na1^iii^—Na1—O6—Co1	−112.70 (8)
O3^i^—Co1—O1—P1	−96.88 (14)	O3^i^—Co1—O7—Na1	−154.99 (11)
O2^ii^—Co1—O1—P1	11.0 (5)	O2^ii^—Co1—O7—Na1	115.12 (11)
O4—Co1—O1—P1	1.70 (13)	O4—Co1—O7—Na1	52.9 (6)
O7—Co1—O1—P1	174.31 (13)	O1—Co1—O7—Na1	−61.95 (11)
O6—Co1—O1—P1	95.95 (14)	O6—Co1—O7—Na1	23.32 (10)
O1—P1—O2—Co1^iv^	−157.24 (16)	O9—Na1—O7—Co1	−19.2 (3)
O3—P1—O2—Co1^iv^	−25.7 (2)	O10—Na1—O7—Co1	−116.96 (11)
C1—P1—O2—Co1^iv^	91.3 (2)	O9^iii^—Na1—O7—Co1	66.71 (11)
O1—P1—O3—Co1^i^	−43.0 (2)	O8—Na1—O7—Co1	146.19 (10)
O2—P1—O3—Co1^i^	−174.31 (17)	O6—Na1—O7—Co1	−20.70 (9)
C1—P1—O3—Co1^i^	72.1 (2)	Na1^iii^—Na1—O7—Co1	45.96 (16)
O3^i^—Co1—O4—C1	89.9 (2)	O10—Na1—O9—Na1^iii^	−178.55 (11)
O2^ii^—Co1—O4—C1	179.2 (2)	O9^iii^—Na1—O9—Na1^iii^	0.0
O1—Co1—O4—C1	−2.5 (2)	O7—Na1—O9—Na1^iii^	84.4 (2)
O7—Co1—O4—C1	−118.4 (6)	O8—Na1—O9—Na1^iii^	−82.39 (9)
O6—Co1—O4—C1	−89.3 (2)	O6—Na1—O9—Na1^iii^	85.74 (9)
O3^i^—Co1—O6—Na1	−13.2 (4)	Co1—O4—C1—O5	178.7 (2)
O2^ii^—Co1—O6—Na1	−110.39 (9)	Co1—O4—C1—P1	2.6 (3)
O4—Co1—O6—Na1	163.11 (8)	O1—P1—C1—O5	−177.4 (2)
O1—Co1—O6—Na1	79.78 (9)	O2—P1—C1—O5	−57.9 (3)
O7—Co1—O6—Na1	−20.89 (9)	O3—P1—C1—O5	60.1 (3)
O9—Na1—O6—Co1	−159.41 (9)	O1—P1—C1—O4	−1.1 (3)
O10—Na1—O6—Co1	107.37 (10)	O2—P1—C1—O4	118.4 (2)
O9^iii^—Na1—O6—Co1	−66.65 (10)	O3—P1—C1—O4	−123.7 (2)

Symmetry codes: (i) −*x*, −*y*+1, −*z*+1; (ii) −*x*, *y*+1/2, −*z*+3/2; (iii) −*x*+1, −*y*+1, −*z*+2; (iv) −*x*, *y*−1/2, −*z*+3/2.

### Hydrogen-bond geometry (Å, º)

**Table:**

*D*—H···*A*	*D*—H	H···*A*	*D*···*A*	*D*—H···*A*
O10—H10*B*···O4^v^	0.82 (1)	1.99 (1)	2.805 (3)	173 (4)
O10—H10*A*···O5^ii^	0.82 (1)	1.99 (3)	2.736 (3)	151 (5)
O9—H9*B*···O1^iii^	0.82 (1)	1.87 (1)	2.683 (3)	178 (4)
O9—H9*A*···O5^vi^	0.82 (1)	1.98 (1)	2.790 (4)	171 (4)
O8—H8*B*···O4^vii^	0.82 (1)	2.24 (2)	2.988 (4)	152 (3)
O8—H8*A*···O2^iii^	0.82 (1)	1.94 (1)	2.751 (3)	168 (4)
O7—H7*B*···O10^viii^	0.82 (1)	1.88 (1)	2.703 (4)	177 (4)
O7—H7*A*···O5^ii^	0.82 (1)	1.96 (2)	2.757 (3)	166 (5)
O6—H6*B*···O8^iii^	0.82 (1)	2.02 (2)	2.806 (3)	161 (4)
O6—H6*A*···O3^ii^	0.82 (1)	1.97 (2)	2.698 (3)	148 (4)

Symmetry codes: (ii) −*x*, *y*+1/2, −*z*+3/2; (iii) −*x*+1, −*y*+1, −*z*+2; (v) *x*+1, −*y*+3/2, *z*+1/2; (vi) −*x*, −*y*+1, −*z*+2; (vii) *x*+1, *y*, *z*; (viii) *x*, −*y*+3/2, *z*−1/2.

## References

[bb1] Galanski, M., Slaby, S., Jakupec, M. A. & Keppler, B. K. (2003). *J. Med. Chem.* **46**, 4946–4951. 10.1021/jm0308040 14584945

[bb2] Jacobson, R. (1998). *REQAB* Private communication to the Rigaku Corporation, Tokyo, Japan.

[bb3] Margiotta, N., Ostuni, R., Teoli, D., Morpurgo, M., Realdon, N., Palazzo, B. & Natile, G. (2007). *Dalton Trans.* pp. 3131–3139. 10.1039/b705239a 17637988

[bb4] Mesri, E. A., Cesarman, E., Arvanitakis, L., Rafii, S., Moore, M. A., Posnett, D. N., Knowles, D. M. & Asch, A. S. (1996). *J. Exp. Med.* **183**, 2385–2390. 10.1084/jem.183.5.2385 PMC2192551 8642350

[bb5] Rigaku (1999). *CrystalClear* Rigaku Corporation, Tokyo, Japan.

[bb6] Rigaku/MSC & Rigaku (2000). *CrystalStructure* Rigaku/MSC, The Woodands, Texas, USA, and Rigaku Coporation, Tokyo, Japan.

[bb7] Sheldrick, G. M. (2008). *Acta Cryst.* A**64**, 112–122. 10.1107/S0108767307043930 18156677

[bb8] Torres Martin de Rosales, R., Finucane, C., Mather, S. J. & Blower, P. J. (2009). *Chem. Commun.* **32**, 4847–4849. 10.1039/b908652h PMC7116767 19652801

[bb9] Westrip, S. P. (2010). *J. Appl. Cryst.* **43**, 920–925.

[bb10] Xue, Z., Lin, M., Zhu, J., Zhang, J., Li, Y. & Guo, Z. (2010). *Chem. Commun.* **46**, 1212–1214. 10.1039/b922222g 20449253

